# Correction: Ashby et al. Femoral Structure and Biomechanical Characteristics in Sanfilippo Syndrome Type-B Mice. *Int. J. Mol. Sci.* 2023, *24*, 13988

**DOI:** 10.3390/ijms26157442

**Published:** 2025-08-01

**Authors:** Frederick James Ashby, Evelyn J. Castillo, Yan Ludwig, Natalia K. Andraka, Cong Chen, Julia C. Jamieson, Nadia Kabbej, John D. Sommerville, Jose I. Aguirre, Coy D. Heldermon

**Affiliations:** 1 Department of Medicine, University of Florida, Gainesville, FL 32611, USA; yludwig@ufl.edu (Y.L.); natalia.andraka@medicine.ufl.edu (N.K.A.); juliajamieson@ufl.edu (J.C.J.); nadia.kabbej@medicine.ufl.edu (N.K.); johnsommerville@ufl.edu (J.D.S.); coy.heldermon@medicine.ufl.edu (C.D.H.); 2 Department of Physiological Sciences, University of Florida, Gainesville, FL 32611, USA; evelynjcastillo@ufl.edu (E.J.C.); aguirrej@ufl.edu (J.I.A.); 3 Department of Orthopaedic Surgery & Sports Medicine, University of Florida, Gainesville, FL 32611, USA

In the original publication [1], there was a mistake in Figure 7 as published. Figure 7 panel c is the same as panel a and did not contain information on ultimate stress. The corrected Figure 7 appears below. The authors state that the scientific conclusions are unaffected. This correction was approved by the Academic Editor. The original publication has also been updated.

## Figures and Tables

**Figure 7:**
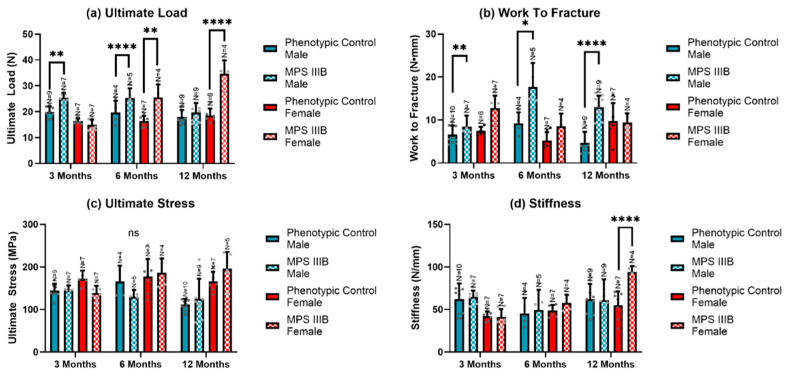
The biomechanical properties of an MPS IIIB mouse femur subjected to a 3-point bend axial stress test from 3 to 12 months of age. Ultimate load (**a**), work to fracture (**b**), ultimate stress (**c**), and stiffness (**d**) are shown (N = 4–10 per group). Statistically significant differences were noted for some groups, particularly ultimate stress for both sexes and work to fracture for males. A two-way ANOVA was performed on each biomechanical property. Post hoc analysis was performed with a Dunn–Šidák correction. Error bars show the standard deviation. * *p* < 0.05; ** *p* < 0.01; **** *p* < 0.0001, ns = not significant.
